# Two-sample Mendelian randomization analysis of 91 circulating inflammatory protein levels and meningioma

**DOI:** 10.1097/MD.0000000000044477

**Published:** 2025-09-26

**Authors:** Jiewen Xin, Qiangbin Zhu, Xianyong Chen, Peikun Huang, Weipeng Hu, Zhenyong Huang

**Affiliations:** a Department of Neurosurgery, Hui’an County Hospital and Hui’an County Hospital Affiliated to Quanzhou Medical College, Quanzhou, China; b Department of Neurosurgery, The Second Affiliated Hospital of Fujian Medical, Quanzhou, Fujian Province, China.

**Keywords:** circulating inflammatory proteins, Fms-related tyrosine kinase 3 ligand, immune inflammation, Mendelian randomization, meningiomas

## Abstract

Meningiomas are prevalent central nervous system tumors with unclear etiological mechanisms. Due to their location outside the blood–brain barrier, they may be susceptible to systemic inflammatory responses. This study aimed to explore the causal relationship between 91 inflammatory proteins and meningiomas through 2-sample Mendelian randomization (MR). Using genome-wide association studies data from 14,824 participants, single nucleotide polymorphisms associated with inflammatory proteins were selected as instrumental variables. Meningioma data were obtained from a Finnish database (1316 cases and 3,13,392 controls). The inverse-variance weighted method assessed associations, with sensitivity analyses to ensure robustness. Reverse MR examined meningioma impact on inflammatory proteins. Forward MR analysis showed elevated Fms-related tyrosine kinase 3 ligand (FLT3L) levels correlated with reduced meningioma risk (OR = 0.72, 95% CI = 0.61–0.85, *P* < .001). Reverse MR suggested meningiomas might lower plasma FLT3L, C-X-C motif chemokine 11, adenosine deaminase, and hepatocyte growth factor levels. FLT3L may play a protective role in meningiomas, warranting further research on inflammatory proteins for prevention and treatment strategies.

## 1. Introduction

Meningiomas arise from the tissues covering the brain’s surface, including arachnoid cap cells and the pia mater’s cellular components.^[1,2]^ These tumors represent a primary form of central nervous system tumor, with an incidence rate of 36.8%.^[3]^ Meningiomas typically exhibit slow progression and are often discovered incidentally during the diagnosis of unrelated conditions.^[4]^ Although they are generally histologically benign, their invasive growth imparts certain malignant characteristics of malignant tumors.^[5]^ Compared to malignant central nervous system tumors, the etiological mechanisms of meningiomas remain less well understood.^[4]^ Since most meningiomas are located outside the blood–brain barrier, they are more vulnerable to systemic immune and inflammatory responses.^[5]^

Inflammation is a fundamental pathological process primarily characterized by a defensive response that occurs when biological tissues are stimulated by damaging factors such as trauma or infection. Excessive inflammation can lead to diseases like sepsis, autoimmune diseases, and atherosclerosis.^[6,7]^ Research has demonstrated that inflammation also plays a significant role in promoting tumorigenesis and tumor progression.^[8–10]^

Mendelian randomization (MR) uses genetic variants strongly associated with an exposure factor as IVs to evaluate the causal relationship between the exposure factor and the outcome.^[11]^ This method addresses a key limitation of observational studies by mitigating the influence of unmeasured confounding factors and reverse causality, thereby providing a more robust approach to causal inference.^[12]^ Given the unclear etiological factors of meningiomas, the authors employed MR to analyze the causal relationship between 91 circulating inflammatory proteins and meningiomas.

## 2. Materials and methods

**Figure 1.**
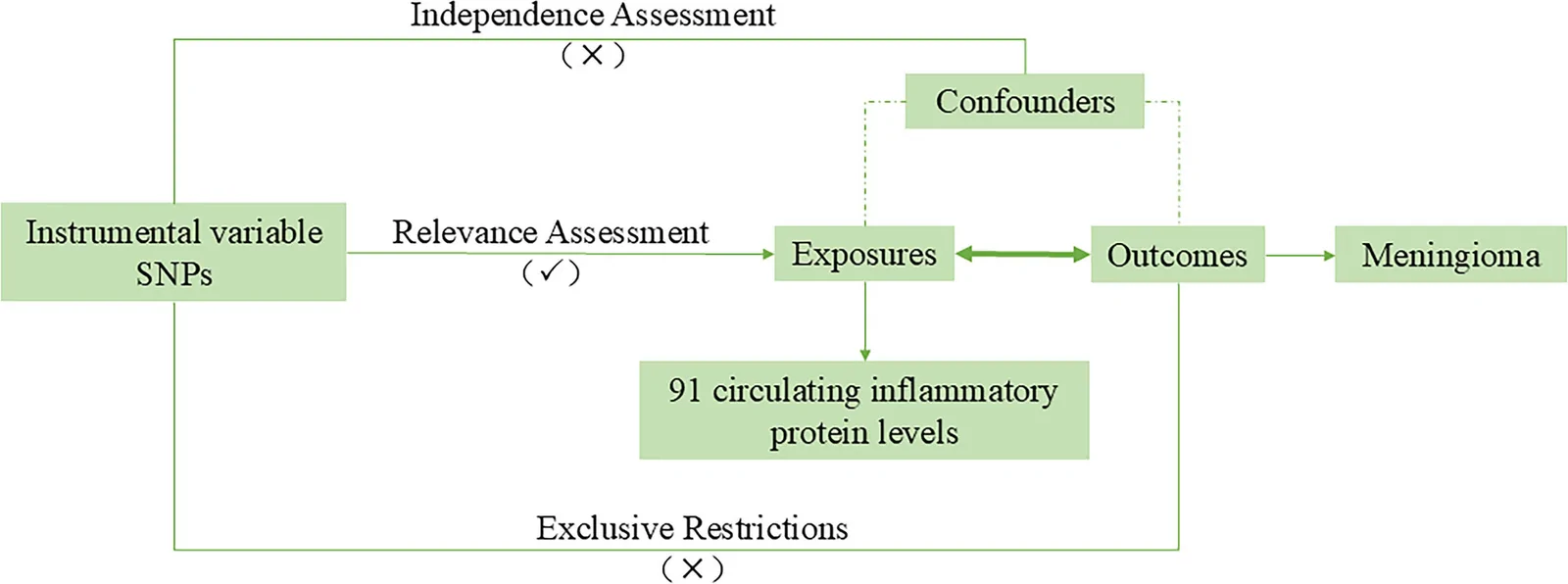
Flowchart figure. SNP = single nucleotide polymorphism.

The research process is illustrated in the flowchart figure (Fig. 1).

### 2.1. Data sources

The summary data for circulating inflammatory proteins were used to select the instrumental variables (IVs) from a genome-wide association studies (GWAS) study that included 14,824 participants.^[13]^ The summary genetic data on meningiomas were obtained from the Finnish R10 database, which includes 1316 meningioma cases and 3,13,392 European-ancestry controls (https://storage.googleapis.com/finngen-public-data-r10/summary_stats/finngen_R10_C3_MENINGIOMA_EXALLC.gz).

### 2.2. Statistical analysis

To evaluate the associations between 91 circulating inflammatory proteins and meningioma, all statistical analyses were performed using R software (version 4.4.1), with the inverse-variance weighted (IVW) method employed as the primary approach. The IVW method is regarded as the most accurate technique for causal estimation when no significant horizontal pleiotropy is present (MR-Egger intercept *P* > .05).

During the selection of instrumental variables (IVs), genetic variants – specifically single nucleotide polymorphisms (SNPs) – that were significantly associated with the exposure and/or outcome variables were selected based on the following criteria. First, to ensure a strong association with the exposure or outcome, only SNPs that reached genome-wide significance (*P* < 5 × 10^−^⁸) in GWAS were included. Second, IV strength was assessed using the *F*-statistic from the first-stage regression, and only SNPs with *F* > 20 were retained to minimize weak instrument bias. Additionally, to ensure independence among IVs, linkage disequilibrium (LD) pruning was conducted: SNPs located on the same chromosome within 10,000 base pairs (clump_kb = 10,000) were considered potentially linked; if the LD coefficient (*r*²) exceeded 0.001 (clump_*r*^2^ = 0.001), only the SNP with the strongest association to the exposure was retained. Following these filtering steps, *F*-statistics were recalculated to confirm IV strength, and only those meeting the threshold (*F* > 20) were included in the MR analysis.

Subsequently, analytical models were chosen based on heterogeneity test results: random-effects models were applied when no significant heterogeneity was detected (MR heterogeneity *P* > .05); otherwise, fixed-effects models were used. The IVW method remained the principal approach for causal inference, supplemented by MR-Egger, weighted median, simple mode, and weighted mode methods to enhance robustness. To ensure result reliability, several sensitivity analyses were performed: heterogeneity among IVs was assessed using Cochran’s *Q* statistic; horizontal pleiotropy was evaluated via MR-Egger, followed by MR-PRESSO global testing and correction for outliers if needed; and leave-one-out analysis was conducted to examine the influence of individual SNPs on the overall estimates.

### 2.3. Multiple testing correction

To balance false-positive control with statistical power, we employed the following approaches:

- False discovery rate (FDR) correction: The Benjamini-Hochberg method was applied to control the false discovery rate (FDR < 0.05), facilitating efficient identification of potentially associated proteins.
- Bonferroni correction: To stringently validate key signals, the Bonferroni-corrected significance threshold was set at α = 0.05/91 ≈ 0.00055. An original *P*-value below this threshold was considered statistically significant. Results are reported as adjusted *P*-values (*P*_Bonferroni = *P* × 91), with *P*_Bonferroni < .05 regarded as significant.

Strong evidence is established only when protein associations are significant under both FDR and Bonferroni corrections. Findings significant solely by FDR should be interpreted with caution and require further validation in subsequent studies.

## 3. Results

After performing correlation analysis, eliminating LD, and excluding weak IVs, eligible single-nucleotide polymorphisms (SNPs) were selected as instrumental variables (IVs). Mendelian randomization (MR) analysis was primarily conducted using the inverse variance weighted (IVW) method as the main analytical approach. To ensure the robustness and reliability of the results, 4 complementary methods-MR-Egger regression, weighted median, simple mode, and weighted mode-were also employed. As a result, 4 proteins (Fms-like tyrosine kinase 3 ligand, C-X-C motif chemokine ligand 11, adenosine deaminase, and HGF) were identified as significantly associated with the risk of meningioma.

### 3.1. Forward Mendelian randomization analysis

**Figure 2.**
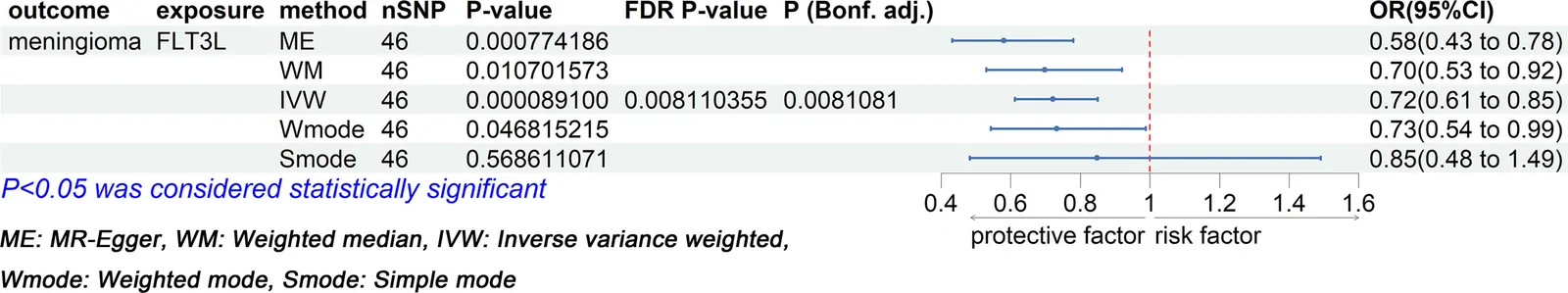
The forest plot for the causal effect of FLT3L on Meningioma by each common MR analytical methods. Significant findings are denoted by *P*(IVW) < .05. FLT3L = Fms-related tyrosine kinase 3 ligand, IVW = inverse-variance weighted, MR = Mendelian randomization.

Using the concentrations of 91 circulating inflammatory proteins as exposures and meningioma as the outcome, causal relationships were assessed. The results demonstrated that higher levels of Fms-like tyrosine kinase 3 ligand (FLT3L) were significantly associated with a reduced risk of meningioma (IVW odds ratio = 0.720; 95% CI: 0.610–0.850; *P* < .001), suggesting a potential protective role of *FLT3L* (Fig. 2 and Supplemental Digital Contents 1–4, Supplemental Digital Content, https://links.lww.com/MD/Q36). The heterogeneity test showed no significant variability among the IVs (*P* = .2817), and MR-Egger regression indicated no evidence of horizontal pleiotropy (*P* = .0862), supporting the robustness of the model’s results. FLT3L is the only protein that remains significant after both Bonferroni correction (FDR-adjusted *P* = .008) and FDR correction (Bonferroni-adjusted *P* = .008; Table 1). Full results for all tested exposures are provided in Supplemental Digital Content 5, Supplemental Digital Content, https://links.lww.com/MD/Q37.

**Table 1:** Forward Mendelian randomization analysis of meningioma on FLT3L (significant threshold: *P*(IVW) < .05).

	Forward Mendelian randomization analysis
Outcome	Meningioma
Exposure	FLT3L
nsnp	46
b-inverse variance weighted	−0.32724
SE-inverse variance weighted	0.083514
*P* value-inverse variance weighted	8.91E–05
FDR-inverse variance weighted	0.00811
Estimate-inverse variance weighted	0.72 (0.61–0.85)
Steiger-*P* value	0
*F*	35.94363
*R* ^2^	0.116965
*Q*-inverse variance weighted	49.99233
*Q*-df-inverse variance weighted	45
*Q*-*P* value-inverse variance weighted	.281744
Egger-intercept	0.027418
SE-Egger	0.015619
*P* value-Egger	.086159
Global-test-*P*	.285
Adjusted-*P* value-Bonferroni	.008108

FLT3L = Fms-like tyrosine kinase 3 ligand, SE = standard error.

### 3.2. Reverse Mendelian randomization analysis

**Figure 3.**
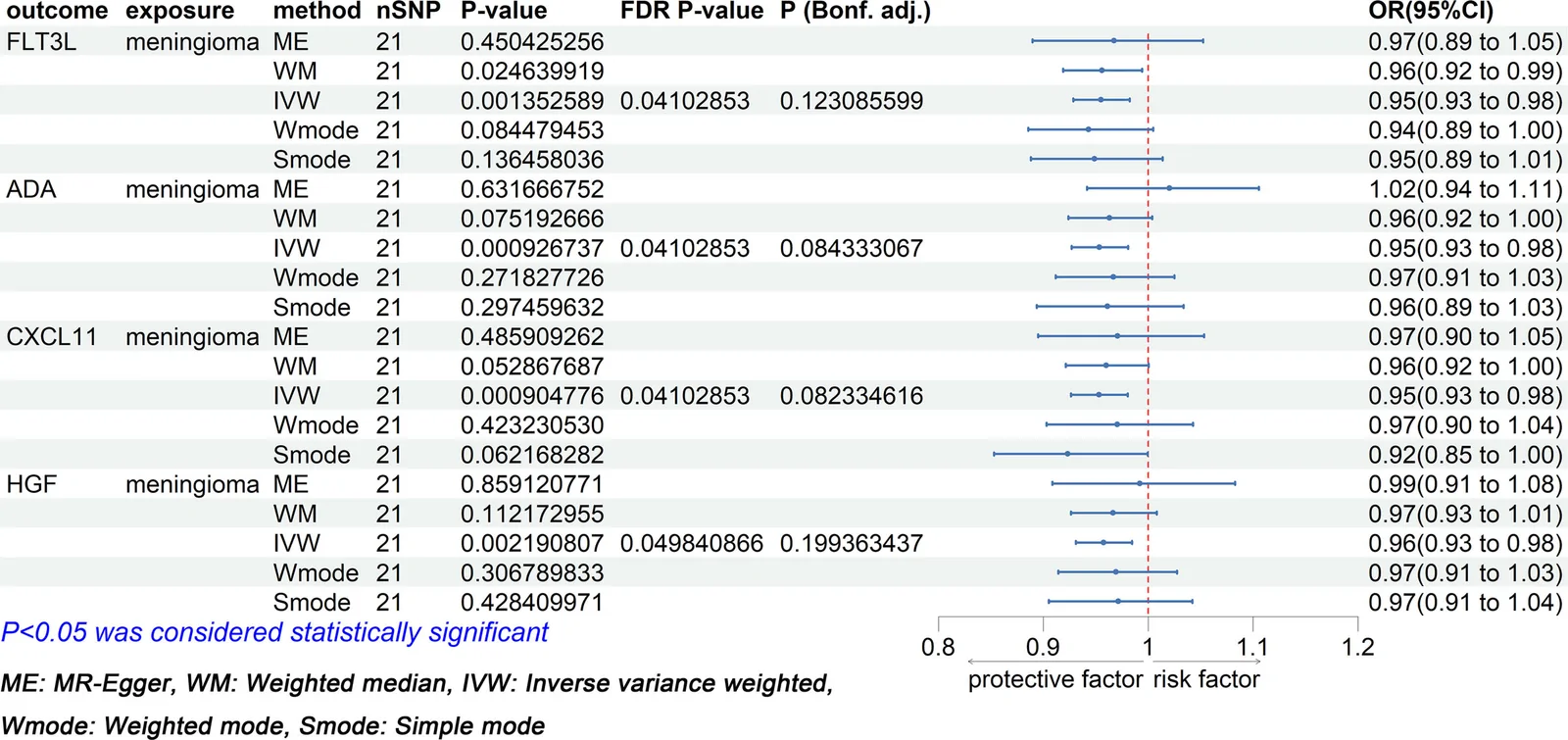
Reverse MR Results. Mendelian randomization analysis with meningioma as the exposure factor and circulating inflammatory proteins as the outcome, considering significance with *P*(IVW) < .05. IVW = inverse-variance weighted, MR = Mendelian randomization.

To investigate potential bidirectional effects, we performed reverse MR analysis using meningioma as the exposure and inflammatory protein levels as outcomes (Fig. 3). Full results for all tested exposures are provided in Supplemental Digital Content 6, Supplemental Digital Content, https://links.lww.com/MD/Q37. Key findings include (Table 2):

**Table 2:** Reverse Mendelian randomization analysis of meningioma on CXCL11, ADA, FLT3L, and HGF (significant threshold: *P*(IVW) < .05).

	Reverse Mendelian randomization analysis			
Outcome	CXCL11	ADA	FLT3L	HGF
Exposure	Meningioma	Meningioma	Meningioma	Meningioma
nsnp	21	21	21	21
b-inverse variance weighted	−0.048	−0.0476	−0.04608	−0.04357
SE-inverse variance weighted	0.014464	0.014373	0.014379	0.014223
*P* value-inverse variance weighted	.000905	.000927	.001353	.002191
FDR-inverse variance weighted	0.041029	0.041029	0.041029	0.049841
Estimate-inverse variance weighted	0.95 (0.93–0.98)	0.95 (0.93–0.98)	0.95 (0.93–0.98)	0.96 (0.93–0.98)
Steiger-*P* value	.278395	.160863	.228803	.175313
*F*	23.8911	23.8911	23.8911	23.8911
*R* ^2^	0.001594	0.001594	0.001594	0.001594
*Q*-inverse variance weighted	22.23614	25.24247	23.74633	27.16077
*Q*-df-inverse variance weighted	20	20	20	20
*Q*-*P* value-inverse variance weighted	.327836	.192312	.253649	.130801
Egger-intercept	−0.00558	−0.02043	−0.00394	−0.01072
SE-Egger	0.011617	0.011476	0.01199	0.012516
*P* value-Egger	.636295	.091082	.745744	.402337
Global-test-*P*	.344	.232	.281	.16
Adjusted-*P* value-Bonferroni	.082335	.084333	.123086	.199363

ADA = adenosine deaminase, CXCL11 = C-X-C motif chemokine ligand 11, FLT3L = Fms-like tyrosine kinase 3 ligand, HGF = hepatocyte growth factor, SE = standard error.

FLT3L: Meningioma showed significant negative associations with Fms-like tyrosine kinase 3 ligand levels (IVW OR = 0.950, 95% CI: 0.930–0.980; *P* < .01), with no heterogeneity (*P* = .25) or horizontal pleiotropy (*P* = .75).

C-X-C motif chemokine ligand 11 (CXCL11): Meningioma status correlated with reduced *CXCL11* levels (IVW OR = 0.950, 95% CI: 0.930–0.980; *P* < .001), showing no heterogeneity (*P* = .33) or pleiotropy (*P* = .64).

Adenosine deaminase (ADA): Adenosine deaminase levels were inversely associated with meningioma (IVW OR = 0.950, 95% CI: 0.930–0.980; *P* < .001), with no significant heterogeneity (*P* = .19) or pleiotropy (*P* = .09).

Hepatocyte growth factor (HGF): HGF levels decreased with meningioma development (IVW OR = 0.960, 95% CI: 0.930–0.980; *P* < .01), with no detectable heterogeneity (*P* = .13) or pleiotropy (*P* = .40).

No proteins passed the Bonferroni correction in the reverse MR analysis; however, 4 proteins (FLT3L, CXCL11, ADA, and HGF) were significant after FDR correction.

These results suggest bidirectional relationships between meningioma and specific inflammatory mediators, strengthening evidence for their potential causal roles in disease pathogenesis.

## 4. Discussion

Meningiomas are a common central nervous system tumor, yet their pathogenesis remains incompletely understood. Due to their asymptomatic nature, meningiomas are often discovered incidentally during examinations for unrelated conditions.^[4]^ Furthermore, their insidious onset complicates early prevention efforts. In this study, the authors employed MR to investigate the causal relationship between 91 circulating inflammatory proteins and meningiomas. The findings revealed that elevated levels of FLT3L were significantly associated with a reduced risk of meningiomas (OR = 0.72, 95% CI = 0.61–0.85, *P* < .001).

Full-length FLT3L is a type I transmembrane protein composed of 231 amino acids in mice and 235 in humans. It consists of an NH₂-terminal signal peptide, followed by an extracellular domain, a transmembrane domain, and a cytoplasmic tail.^[14]^ The Fms-like tyrosine kinase 3 (FLT3) receptor is a key regulator of dendritic cell (DC) development and plays a critical role in controlling the differentiation of DCs at various stages.^[15]^ Under steady-state conditions, the interaction between FLT3L and FLT3 induces the generation of conventional dendritic cells (cDCs) and plasmacytoid dendritic cells.^[16]^ cDCs contribute to the induction of adaptive immunity and tolerance. DCs regulate immune responses by inducing apoptosis of inflammatory T-cells, restoring immune homeostasis through modulation of pro-inflammatory and anti-inflammatory responses, and amplifying regulatory T-cells.^[17,18]^ The extent of immune cell infiltration influences the development and progression of meningiomas.^[19]^ DCs are located in the meninges and choroid plexus,^[20]^ and cDCs are involved in the antitumor immune processes of brain tumors, while brain tumors stimulate the expansion of FLT3L-sensitive DC populations.^[21]^ Recent studies suggest that FLT3L enhances the efficacy of antitumor drugs by indirectly modifying the tumor microenvironment through the induction of DC differentiation.^[22]^ In this study, high levels of FLT3L were associated with a reduced risk of meningiomas, and reverse MR analysis indicated that the occurrence of meningiomas leads to a decrease in plasma FLT3L levels (OR = 0.95, 95% CI = 0.93–0.98, *P* < .01). FLT3L may act as an antitumor factor, likely due to its involvement in regulating DC differentiation, which indirectly influences antitumor immunity.

CXCL11 also known as IFN-inducible T-cell alpha chemokine, is primarily secreted by monocytes, endothelial cells, fibroblasts, and cancer cells in response to interferon.^[23,24]^ CXCL11 exhibits diverse roles in various tumor types, acting both to promote tumor progression and to exert antitumor effects.^[24]^ Recent studies have suggested that CXCL11 may contribute to the progression and spread of meningioma cells.^[25]^ In this study, the occurrence of meningiomas was associated with decreased plasma levels of CXCL11 (OR = 0.95, 95% CI = 0.93–0.98, *P *< .001). However, the specific role of CXCL11 in meningiomas warrants further investigation.

ADA is a key enzyme involved in purine metabolism. It is a 41 kDa monomeric protein that serves as a catalyst, allosteric regulator, and intercellular communication molecule.^[26]^ Previous studies have reported no significant changes in serum ADA levels in patients with central nervous system tumors.^[27]^ However, the present analysis found that meningioma exposure was associated with decreased plasma ADA levels. Recent studies suggest that ADA levels vary across different tumor types, and targeting purine metabolism may offer therapeutic potential for nervous system tumors.^[28]^

HGF also known as scatter factor, is primarily secreted by mesenchymal cells. It is a polypeptide growth factor belonging to the plasminogen family and plays a role in regulating immunity and inflammation.^[29]^ HGF may serve as a protective factor by alleviating inflammatory responses through the inhibition of the NF-κB pathway.^[30,31]^ Additionally, HGF regulates the migration, activation, and immune-stimulatory functions of DCs.^[29,32,33]^ The present analysis found that meningioma exposure was associated with decreased plasma HGF levels, suggesting that the occurrence of meningiomas may compromise HGF’s protective role in immune and inflammatory regulation.

In the forward MR analysis, FLT3L was the only circulating inflammatory protein that remained statistically significant after both Bonferroni and FDR correction, suggesting a potential causal association between elevated plasma FLT3L levels and reduced risk of meningioma. This finding was consistent across various MR methods and sensitivity analyses, and aligns with the proposed role of FLT3L in promoting antitumor immune responses.

In contrast, the reverse MR analysis identified FLT3L, CXCL11, ADA, and HGF as potential proteins causally influenced by meningioma, based on FDR correction. However, the statistical evidence for these associations was relatively limited, with FDR-adjusted *P*-values approaching .05 and no significance under Bonferroni correction. Given the lack of direct experimental or observational evidence linking meningioma exposure to changes in CXCL11, ADA, or HGF levels, our MR findings for these proteins should be considered exploratory and interpreted with appropriate caution. These proteins may serve as prioritized candidates for future validation through experimental approaches or larger-scale MR analyses.

It is important to note that the stringency of Bonferroni correction may obscure some true associations, whereas FDR correction offers a more balanced approach between controlling false positives and retaining statistical power. Therefore, we recommend interpreting these findings with caution, taking into account the results from both correction methods.

## 5. Limitations

A potential limitation of this study is the possible sample overlap between the GWAS datasets for circulating inflammatory proteins and meningioma risk in the FinnGen R10 cohort. In particular, the protein GWAS partially included Finnish cohorts such as FINRISK, which may overlap with the FinnGen population. Such overlap can introduce bias in 2-sample MR analyses, potentially inflating causal estimates when instruments are weak. Although the *F*-statistics indicated acceptable instrument strength, shared participants may still affect the reliability of our findings. Therefore, the results should be interpreted with caution, and replication in fully independent datasets is warranted.

In addition to sample overlap, another important consideration is the ancestry composition of the study population. The study population is predominantly composed of individuals of European ancestry, which may limit the generalizability of our findings to other populations. To mitigate potential biases arising from ancestral differences, future research should broaden the population scope.

## 6. Conclusion

In conclusion, bidirectional MR analysis reveals a potential reciprocal relationship between FLT3L and the risk of meningioma. Elevated circulating FLT3L levels may reduce the risk of developing meningioma (forward MR), whereas the presence of meningioma may lead to decreased FLT3L levels (reverse MR). This bidirectional pattern suggests that FLT3L functions both as a protective factor and as a regulatory target within the tumor microenvironment. Furthermore, tumor-driven alterations in other circulating proteins, such as CXCL11 and HGF, may provide novel insights for diagnostic strategies. Further clinical studies are warranted to validate these findings and clarify the underlying biological mechanisms. These proteins may represent promising targets for the prevention, diagnosis, and treatment of meningiomas.

## Author contributions

**Conceptualization:** Jiewen Xin, Qiangbin Zhu, Xianyong Chen, Weipeng Hu.

**Data curation:** Qiangbin Zhu, Peikun Huang, Weipeng Hu, Zhenyong Huang.

**Investigation:** Qiangbin Zhu, Xianyong Chen, Weipeng Hu.

**Methodology:** Peikun Huang.

**Resources:** Zhenyong Huang.

**Supervision:** Weipeng Hu, Zhenyong Huang.

**Writing – original draft:** Jiewen Xin, Qiangbin Zhu, Xianyong Chen.

**Writing – review & editing:** Jiewen Xin, Weipeng Hu, Zhenyong Huang.

## Supplementary Material
