# Supplementary material for: Two-sample Mendelian randomization analysis of 91 circulating inflammatory protein levels and meningioma
Source: Medicine (Baltimore). 2025 Sep 26;104(39):e44477. doi: 10.1097/MD.0000000000044477 (PMC13593242; doi:10.1097/MD.0000000000044477)
Supplement: Supplementary file 1 [file medi-104-e44477-s001.docx]

**Supplemental Digital Content 1** Forest plot of the causality of FLT3L on meningioma.

**Supplemental Digital Content 2** Leave-one-out analysis of the causality of FLT3L on meningioma.

**Supplemental Digital Content 3** Funnel plot of the causality of FLT3L on meningioma.

**Supplemental Digital Content 4** Scatterplot of the causality of FLT3L on meningioma (exposure: FLT3L; outcome: meningioma). MR, Mendelian randomization; SNP, single nucleotide polymorphism.
